# Restricting Sales of Menthol Tobacco Products: Lessons Learned from Policy Passage and Implementation in Minneapolis, St. Paul, and Duluth, Minnesota

**DOI:** 10.1089/heq.2020.0137

**Published:** 2021-06-18

**Authors:** Linda M. Bosma, Joanne D'Silva, Joanne Moze, Chris Matter, John H. Kingsbury, Betsy Brock

**Affiliations:** ^1^ Bosma Consulting, LLC, Eagan, Minnesota, USA.; ^2^ ClearWay Minnesota, Minneapolis, Minnesota, USA.; ^3^ Center for Prevention, Blue Cross and Blue Shield of Minnesota, Eagan, Minnesota, USA.; ^4^ Minnesota Department of Health, Saint Paul, Minnesota, USA.; ^5^ Association for Nonsmokers-Minnesota, Saint Paul, Minnesota, USA.

**Keywords:** menthol and flavor tobacco policy, tobacco control, implementation, health disparities

## Abstract

**Purpose:** Commercial tobacco products have historically been target marketed to African American, Latinx, Asian American Pacific Islander, Indigenous, and Lesbian, Gay, Bisexual, Transgender, Queer (LGBTQ) communities, as well as to youth. Menthol cigarettes increase smoking initiation and decrease smoking cessation, particularly among African Americans who smoke menthol cigarettes at higher rates than their white peers. Due to disproportionate tobacco-related health consequences for members of these communities, effective tobacco control policies that restrict availability of menthol products by focusing on retail sales are an important element of addressing health disparities, and require policy efforts informed by leadership and the voice of communities most impacted. This study examines the organizing efforts of three successful policy initiatives in 2017–2018 in Minneapolis, St. Paul, and Duluth, Minnesota, and identifies facilitators and barriers of these campaigns.

**Methods:** We conducted 50 key informant interviews with city council/staff, advocates, and community members and analyzed them for emerging themes. The analysis employed a process-oriented qualitative matrix process to identify emerging themes and divergent perspectives.

**Results:** Following policy implementation, outlets selling commercial menthol tobacco products substantially decreased. Facilitators included strong city council support, leadership from impacted communities, community awareness-building campaigns, and understanding tobacco industry counter-tactics. Challenges included the need to counter tobacco industry misinformation and retailer attempts to circumvent the intent of restrictions.

**Conclusion:** Well-planned advocacy campaigns led by community members most impacted by commercial tobacco can overcome opposition and challenges to restrict sales of menthol tobacco products and successfully reduce availability of these products in their communities.

## Introduction

In 2017–2018, Minneapolis, St. Paul, and Duluth became the first three Minnesota cities to pass policies restricting the sale of menthol-, mint-, and wintergreen-flavored commercial^†^ tobacco products to adult-only tobacco shops,^‡^ and, in Minneapolis and St. Paul, liquor stores. These policies resulted from robust community engagement, policy advocacy, and educational campaigns. These campaigns were led by members of African American, Latinx, Asian American Pacific Islander, Indigenous, and Lesbian, Gay, Bisexual, Transgender, Queer (LGBTQ) communities, which are the communities most impacted by menthol tobacco-related disparities and youth advocates. As interest in policy action on menthol tobacco gains momentum,^1^ more information is needed on elements that contribute to successful campaigns.

Menthol cigarettes are a public health threat because they increase smoking initiation among youth, increase addiction, and decrease smoking cessation, particularly among African Americans who smoke menthol cigarettes at higher rates than their white peers.^2^ Evidence indicates that menthol cigarettes are disproportionately marketed to racial and ethnic minorities, as well as women and youth.^3,4^ Research shows that, for cigarettes, menthol advertising and price promotions are more prevalent in low-income and African American neighborhoods.^5,6^ Restricting the sale of commercial menthol tobacco products is a policy approach that has potential to contribute to reducing the burden of smoking among African Americans and other priority populations.

Menthol is also pervasive in other tobacco products. Kuiper et al.^7^ examined 2015 Nielsen tobacco sales data and found that 19.4% of little cigars, 57.0% of moist snuff, and 88.5% of snus tobacco sales were menthol flavored. Among high school students, during 2017–2018, current use of menthol- or mint-flavored e-cigarettes increased among all current e-cigarette users (from 42.3% to 51.2%, *p*=0.04).^8^ Although the manufacture of fruit- and candy-flavored cigarettes was banned by the 2009 Family Smoking and Prevention Tobacco Control Act due to their appeal to youth, menthol-flavored cigarettes were exempted, but the United States Food and Drug Administration (FDA) was given the power to regulate menthol.^9^ More than a decade later, the FDA has yet to take action on regulating menthol cigarettes.

As a result of FDA inaction, local jurisdictions are enacting policies to restrict the sale of these products. The policies passed in Minnesota were among the first in the country to enact comprehensive restrictions on all menthol tobacco products, including e-cigarettes. Most prior efforts to restrict flavored tobacco products included exemptions for menthol-flavored tobacco products or e-cigarettes.^10^ In 2013, Chicago was the first jurisdiction to take action on menthol products by limiting sales within a geographical radius of schools. Since 2017, other cities, including San Francisco, have been successful banning the sale of all flavored tobacco products, including menthol, at all retail locations. In November 2019, Massachusetts became the first state to ban the sale of flavored tobacco products. When the tobacco industry attempted a referendum to overturn San Francisco's ban on flavored tobacco products in 2018, San Francisco voters upheld the ban on menthol products by a 68% to 32% margin.^11^ As of August 2020, at least 100 municipalities in nine states have enacted restrictions and/or bans on menthol tobacco products.^12^ A growing body of evidence indicates that these policies can be effective in reducing flavored tobacco product availability, decreasing initiation among youth, and encouraging adults to quit.^13–15^

While published evidence usually focuses on evaluating the impact of the policy change, there is also value in examining facilitators and barriers of successful policy campaigns, especially as these policy strategies are relatively nascent compared to other evidence-based tobacco control policy approaches (e.g., smoke-free air and excise taxes). It is especially important to examine policy initiatives that engage in and impact those communities most impacted by commercial tobacco harms and targeted by the tobacco industry to further efforts that address health equity and reduce health disparities. Health equity is both process and outcome; thus, it is important to look at the process that is undertaken to achieve policy initiatives. The purpose of this study was to document characteristics that contributed to successful policy passage and implementation to inform other jurisdictions around the country that may be interested in reducing availability of menthol.

## Methods

We conducted 50 semistructured key informant interviews with a purposive sample of advocates, organizers, public health staff, stakeholders, and elected officials identified by staff at organizations funding the advocacy work and advocacy agencies. Respondents were selected for knowledge of policy passage and implementation efforts. Interviews were supplemented by review of campaign materials, media, and council proceedings. Funding agency leaders participated in the development of interview questions.

Interviews were conducted in two stages: shortly after ordinance passage (January–July 2018) and during implementation (January–September 2019). Policy passage interviews focused on respondents' experience and role in campaigns, challenges, and facilitators of passage, opposition, and resistance from retailers and industry. Implementation interviews focused on implementation experiences, what worked well, challenges, and unintended consequences. Interviews were conducted in-person, with a few exceptions to accommodate schedules and inclement weather, and lasted approximately one hour. Interviews were recorded and transcribed.

The policy passage sample included 35 individuals (8 from Minneapolis, 8 from St. Paul, 4 who participated in both the Minneapolis and St. Paul campaigns, and 15 from Duluth).

The implementation sample consisted of 15 interviews with 13 individuals (6 from Minneapolis, 5 from St. Paul, and 2 from Duluth; 1 interview was a group interview with 3 city compliance staff; and 4 individuals participated in additional follow-up interviews to gather information on ongoing developments in implementation.)

Additional guidance and input into the study were provided by the Minnesota Menthol Evaluation Advisory Group, composed of tobacco control stakeholders from funding organizations, local/state public health staff, community advocates, and partners. They provided input on the evaluation design, questions, identifying interview respondents, and analysis.

The analysis employed a process-oriented qualitative matrix analysis.^16,17^ Emerging themes and divergent perspectives were identified, coded, and categorized in themes by city. Preliminary coding decisions were reviewed in two meetings with Advisory Group members and funding agency staff for validation and omission, and to identify areas for additional investigation. The evaluator conducted two presentations of findings to the Advisory Group for final validation.

The Minnesota Department of Health Institutional Review Board reviewed study protocols and instruments and determined the evaluation was exempt. Before interviews, the evaluator explained the evaluation, purpose, audience, confidentiality, benefits, and risks. Respondents signed a consent form verifying they understood the protocols. The evaluator obtained permission to record interviews from each respondent. Respondents were offered a $25 gift card upon completion of the interview.

## Results

Advocates in each city developed unique organizing campaigns specific to local conditions.^18^ Ordinances in these three cities restricting the sale of menthol tobacco products to adult-only tobacco shops (and liquor stores in Minneapolis and St. Paul) passed between August 2017 and February 2018, with implementation 1 year later in Minneapolis and St. Paul and 4 months after passage in Duluth. Outlets selling menthol tobacco decreased 62%, 76%, and 95%, respectively, in the three cities (Table 1). Several themes were important for advocacy efforts to facilitate a strong campaign and to counter challenges of implementation (Table 2).

**Table 1.** Tobacco Licenses Before and After Ordinances Restricting Menthol

City	Dates of council passage and implementation		No. of outlets allowed to sell menthol		Decline	
	Passed council	Implementation	Before	After	Number	Percent
Minneapolis	August 4, 2017	August 1, 2018	342	82	−260	76
St. Paul	November 1, 2017	November 1, 2018	241	92	−149	62
Duluth	February 12, 2018	June 12, 2018	84	4	−80	95

**Table 2.** Themes of Successful Menthol Policy Advocacy and Implementation, Minneapolis, St. Paul, and Duluth, MN

Theme	Sample quote
1. Respect local context.	We thought about adding menthol to [flavors ordinance], but we hadn't really done the community engagement around that … like we were on the verge of passing the flavor piece, so we didn't want to slow that down, and we didn't want to add it because we hadn't done the proper engagement. So, in retrospect I don't think we could have done it differently because of that. The community right now and most of the communities that are looking at it are looking at the whole package because now the precedence has been set, that it's possible.

2. Build knowledge and capacity.	We had a dynamic speaker…who came and spoke at great length about how our communities, communities of color, were targeted with these ads. It was amazing that I actually, growing up with *Jet* and *Ebony* magazine in our household, could immediately remember those ads.
	I think the fact that we spent 3 years educating the community and engaging the community, especially those impacted, led to a very diverse coalition.
3. Develop education campaigns with community.	We really tried to be very conscious of developing materials, really including people…bringing everybody into the decision-making body.
	We spent quite a lot of time getting materials right. We felt that was really important. We talked to a lot of people. We showed people the concept…We had a good group of people who could give us honest feedback.
4. Campaigns led by impacted communities.	The thing that persuaded me was advocacy for people within the most affected communities. That's what really convinced me, because I tend to be somewhat skeptical of people who advocate on other people's behalf. I'm much more persuaded by people who advocate on their own behalf. So, when it was people from within our local African American community, our local Indigenous community, our Lesbian, Gay, Bisexual, Transgender [Queer] community, which are the targeted communities coming forth on their own, basically saying, ‘We are asking you to protect our kids from the use of this product.’ That's what persuaded me. It wasn't just public health professionals.

5. Address racial inequities head on.	Race was a big issue with menthol because everybody knows that African Americans choose menthol over other flavors. So, it was a conversation you had to have. I would say to people, make sure that the [council] room is not filled with white advocates because elected officials are aware of what communities are going to be most impacted by reduction of sales [of menthol] and they don't want the backlash. I walked in the room talking about it. That was the conversation I wanted to have with people. I'm Black. It's firsthand information coming from me.

6. Frame the issue #1: restrictions focus on sellers, not tobacco users.	People would often ask,‘Are you trying to regulate what adults do? This doesn't seem right.’ And so, then we said, well, no…We want adults who are addicted to have access to the products. We know they're addicted. And they need to figure out how to quit if they can…This is really about the next generation of young people, particularly people of color. Let's not have another generation of people addicted to these products.
7. Frame the issue #2: “people over profits.”	
	If you are also talking about [retailers'] talking points, that is the message that gets across, and not the message you want to get across. And so, what I thought advocates that I saw showing up time and time again for this particular issue do effectively was, not to actually talk about the money piece of it, and actually talk about how it was impacting people's lives.

	Let me share with you what this black woman lost. And I told them to look at [my] poster. It was my mother, three aunts, and others…I said, for me, black lives matter, but apparently to you, black lives don't matter. And I know you're tired of hearing black lives don't matter, but you're going to continue to hear about black lives matter until black lives matter!
8. Anticipate and counter tobacco industry tactics.	If I had any doubt that this policy would reduce nicotine addiction…it was completely resolved by the amount of money and effort the tobacco companies were spending to kill it.
9. No surprises: preparation and training.	The advocates provided really helpful information and research…and figured out what mattered most to individual elected officials.
10. Sustain advocacy to maintain city council support.	They've gone and testified against these store owners who want to quit. I think it's important that you don't just shut the lights off after it passes and go away. There can still be policy issues that come up and you don't want the council to say hey, let's just get rid of this whole thing. You know? So yeah, prepare for opposition and keep your coalition engaged through implementation because you'll probably need them.
11. Collaborate with city staff.	Having [an advocacy organization] as a partner is very beneficial. Anytime government can collaborate with a group that's supportive of something, we're always gonna be more effective. The fact it's community-based also is very beneficial. In terms of when regulations are being proposed, the coalition, they're doing the education with the council and things like that. Once again, government works the best when the community is at the table.
12. Campaigns should be managed by a lead agency with demonstrated cultural competency and strong community organizing and policy capacity.	We have a problem in our communities sometimes where white people actually wanna lead an initiative for people of color. It doesn't work.. . This movement felt like the allies were in complete support, and used their resources to help, but it was the community that was in charge.
	So, I'm saying in short there were a bunch of quality people who behaved out of ethical principles, who kept the prize in sight. Who did not misuse people in their organizing.”

### Facilitators of policy passage

#### Respect local context

At the time Minneapolis and St. Paul coalitions began to consider menthol restrictions in 2015, they already had strong youth-led campaigns underway to address other flavored tobacco products (e.g., fruit and candy flavors). Rather than trying to adjust this campaign to incorporate the social and racial justice messaging specific to menthol products, advocates made a strategic decision to first pass other flavors restrictions, and then return to councils to address menthol products. This enabled them to lay the groundwork for menthol restrictions, begin educating city councils on menthol, and mobilize additional stakeholders. In Duluth, policy work began later, and advocates chose to address all flavors, including menthol, in one policy.

#### Build knowledge and capacity

Local organizers devoted two or more years to educating advocates, community members, decision-makers, and stakeholders on the historical tobacco industry targeting of menthol to African American, American Indian, and LGBTQ communities, as well as youth, and brought in national African American leaders to educate and energize local activists.

#### Develop education campaigns with community

Education campaigns complemented and supported advocacy. Community members developed the Lethal Lure (Duluth; https://www.lethallure.org/) and Beautiful Lie, Ugly Truth (Minneapolis and St. Paul; https://www.beautifullieuglytruth.org/) campaigns reflecting how menthol is targeted to their communities and included branded materials, social media, fliers, and t-shirts that advocates easily identify at events and hearings. When community members testified, they wore campaign t-shirts, making them easily identifiable to city council members and the media.

#### Campaigns led by impacted communities

Organizers recruited and mobilized people from communities targeted by the tobacco industry; individuals and organizations from African American, Latinx, American Indian, Asian American, and LGBTQ communities led the campaigns. As one advocate said, “No decision about us without us.” Representative leadership from impacted communities was essential for educating elected officials who needed to know this campaign was led by impacted community members and not just public health professionals.

#### Address racial inequities head on

Campaigns directly addressed racial targeting in council education efforts. Some elected officials expressed hesitation to address menthol, fearing their efforts might be seen as paternalistic. Therefore, it was essential that members of impacted communities spoke directly to the disproportionate harm that tobacco causes their communities.

#### Frame the issue #1: restrictions focus on sellers, not tobacco users

Advocates anticipated opponents would argue that the ordinance would criminalize African American men. They made clear ordinances focused on retailers, not tobacco users, with the ultimate goal to help prevent young people from starting to use tobacco. Acknowledging that menthol restrictions make it less convenient for adults to purchase them, advocates also provided information on cessation services.

#### Frame the issue #2: “people over profits”

While retailers argued they would lose sales, advocates continually reminded decision-makers the cost to the community was human lives. Community members shared stories—and often photos—of loved ones lost to tobacco-related diseases.

#### Anticipate and counter tobacco industry tactics

Experienced organizers familiar with tobacco industry tactics anticipated and responded to industry strategies that included supporting local retailers and disseminating opposition messages through various channels, including point-of-sale postcards encouraging patrons to call council members, sending mailers to every household in one city, local media buys, and an industry-sponsored forum featuring national spokespeople asserting the ordinances were racist and would increase interactions between Black males and police. Advocates countered opposition messages by exposing industry efforts as disingenuous.

#### No surprises: preparation and training

Organizers trained advocates on working with elected officials and prepared decision-makers with research, fact sheets, and counter-arguments so they could readily speak to concerns and opposition.

### Implementation lessons

Advocates and cities encountered several challenges and unintended consequences as after the policies were passed and retailers attempted to find ways to continue selling menthol products (Table 3).

**Table 3.** Implementation Challenges and Responses from Advocates and Cities

Challenges/unintended consequences	Advocate and city responses
“Store splitting”—dividing a convenience store with a wall, adding a separate exterior entrance, and opening as a tobacco products shop	A moratorium on new tobacco products shop licenses to allow time to study density and location of shops that can sell menthol products
Changing license from a convenience or grocery store to a tobacco product shop	Density studies of current outlets to determine how many council wards have shops, and demographic and income information on location of shops
Store within a store—building a separate structure within a store and calling it a tobacco product shop	Set limits on spacing, requiring at least 2000 feet between tobacco product shops
Posting signs in stores informing customers that menthol can no longer be sold—and advising them to contact their council member	Set cap on the total number of licenses allowed in city
	Clarify ordinance intent with all governing bodies that can approve license changes
	Collaboration with city compliance staff (licensing, zoning, law enforcement, etc.) and assist with retailer education, monitoring, and enforcement
Unsold products on hand—merchants still had menthol products on hand when the ordinance went into effect	

LGBT[Q], Lesbian, Gay, Bisexual, Transgender [Queer].

#### Sustain advocacy to maintain city council support

Monitoring implementation was critical as some retailers attempted to circumvent the intent of newly passed ordinances. It was essential to keep advocates and city councils engaged to support modifications to ensure policies would achieve the intended goal of reducing menthol availability. Policies were strengthened by adding density and spacing limits on tobacco retailers.

#### Collaborate with city staff

Advocates collaborated with city staff who implement the ordinance, researching products that contain menthol, providing merchant education, and creating educational materials.

#### Campaigns should be managed by a lead agency with demonstrated cultural competency and strong community organizing and policy capacity

Experienced tobacco control advocacy organizations with paid staff led menthol policy efforts. All had experience with their city councils and credible reputations in their cities, and were known for maintaining respectful, productive relationships. Since menthol disproportionately impacts African American, American Indian, Latinx, Asian American Pacific Islander, and LGBTQ communities, as well as young people, it was essential these organizations demonstrated cultural competency and were sensitive of privilege, built leadership within impacted communities, and took direction from them.

## Discussion

The ordinances in Minneapolis, St. Paul, and Duluth substantially reduced the number of outlets selling menthol products. An initial study found high rates of compliance.^19^ Reducing availability of tobacco products is associated with reduced prevalence of tobacco use.^20–22^ Since young people often begin tobacco use with menthol or other flavored tobacco products,^23^ reducing their access and exposure to these products is anticipated to reduce tobacco initiation and use.

Skilled community organizing campaigns recruited authentic leaders from communities most impacted by menthol tobacco.^24^ Representative local leadership was persuasive with council Members; constituents advocating on behalf of their communities resonated more than a campaign run by professionals. Tobacco industry targeting of African American, American Indian, Latinx, Asian American Pacific Islander, and LGBTQ communities is well documented.^25,26^ When advocates learned how they were targeted deliberately, they connected to the social justice cause. Bringing in national public health experts to speak to the targeting of African Americans resonated with many in the community, especially those old enough to remember ads for menthol cigarettes in magazines like Ebony and Jet or industry-sponsored Kool Jazz concerts.^27^

Local advocates were aware that the tobacco industry sponsored community meetings in Oakland, California, and other cities enlisting nationally recognized African American spokespeople to question efforts to restrict menthol.^28^ When a similar meeting was held in Minneapolis, local advocates were prepared to effectively rebut charges that restrictions would criminalize Black smokers and create an illicit tobacco market. Advocates pointed out that the ordinance focused on the sellers of tobacco and protected young people, and that little evidence exists of increased illicit tobacco trade related to tobacco control policies.^29^

Sustaining advocacy efforts post-passage was critical as retailers attempted to circumvent the intent of ordinances. Community advocates were often the first to notice efforts to continue menthol sales, since these were stores in their communities. Advocates who had the experience of leading campaigns to pass policy continued their leadership to ensure implementation went as intended. They reminded decision-makers that these policies were designed to protect communities of color to address the years of health disparities caused by marketing menthol products to marginalized communities. Their voices were recognized as authentic because the campaign had been led with their wisdom and experience throughout. It was necessary to strengthen language, capping number of tobacco retail outlets and enacting minimum spacing between retailers. Collaborative relationships with city staff have provided valuable support for enforcement and compliance work.

Menthol campaigns in each city were developed specifically for that city, but it was essential in all three cities to have leadership in the campaign come from the communities most impacted by tobacco harms, and to be able to show evidence of how menthol products are marketed to addict African American, Latinx, Asian American Pacific Islander, American Indian, and LGBTQ communities. Even though all three cities had many elected officials who were supportive generally of public health and tobacco control, they needed the assurance that a policy that would impact priority population communities was led by and supported by them. Likewise, youth engagement was motivating for city leaders. Filling city council chambers with visible advocates who spoke to the cost in lives and health caused by menthol tobacco products was equally important in all three settings. The cities described in this study are the three largest cities in the state and all three campaigns attracted opposition from the tobacco industry and retailers, so organized campaigns that were well resourced were essential.

### Health equity implications

When Minneapolis, St. Paul, and Duluth began their organizing campaigns to enact menthol restrictions, such efforts were in their infancy in the United States. Advocates in Minneapolis and St. Paul had begun working to restrict availability of other flavored tobacco products knowing similar restrictions elsewhere had withstood court challenges. For them, it made sense to adopt restrictions on other flavors first, and then pursue menthol restrictions. Leaders said it would not have worked in their cities to do it any other way when they began their efforts. By the time the city of Duluth considered a ban, they felt confident about tackling all flavors (including menthol) in one ordinance. Their efforts have encouraged other municipalities to undertake menthol restrictions. Other Minnesota cities have adopted similar policies and a statewide sales ban on all flavored tobacco products was introduced in the 2020 Minnesota Legislature.^30^ Cities and states are moving toward comprehensive ban of all flavors in all tobacco products. However, the FDA removed flavored e-cigarette pods and cartridges from the marketplace, but exempted menthol-flavored pods as well as other types of e-cigarette devices such as flavored disposables, which are still available in menthol and other flavors.^31^ This decision highlights the need for ongoing policy-making at the local and state level to protect priority populations, including youth, by closing the loopholes left in place by the FDA.

In Massachusetts, early evidence indicates adolescent cigarette and e-cigarette use decreased 1 year after restrictions on flavored tobacco products went into effect^15^ and a ban in New York City product sales and chances of teen use of any tobacco products declined significantly after enforcement began.^14^ Outside the United States, the European Union banned menthol products in May 2020^32^ and Canada banned menthol as of January 2017, after 7 of its 10 provinces banned menthol sales in the two previous years. Early evidence from Canada is encouraging. Ontario's ban on menthol sales was associated with a significant reduction of menthol cigarette sales and total cigarette sales compared to British Columbia where there was no provincial menthol ban.^33^ Another study found higher rates of quitting smoking among daily and occasional menthol smokers one year after implementation of Ontario's menthol ban compared with non-menthol smokers.^13^ Stoklosa^34^ found no surge in illicit cigarettes after Nova Scotia's 2015 ban on menthol cigarette sales. This suggests restricting availability of menthol tobacco products may lead to improvements in public health. Similar policies in the United States have the potential to reduce health disparities related to targeted marketing of menthol tobacco products to priority populations.

### Limitations and future research

Our study of three Minnesota cities may not be generalizable to other locations since Minnesota has a strong tobacco control movement and as of 2018 has reduced its adult smoking rate to 13.8%,^35^ which may contribute to a more receptive environment for tobacco control. Our sample was limited to stakeholders supportive of the policy; no opponents were interviewed. This was a retrospective study, so there is potential for recall bias. Our focus was policy passage and implementation processes; future research should examine the association between menthol restrictions and tobacco use prevalence. In addition, ongoing monitoring of implementation and compliance is necessary to ensure the policies continue to be enforced as intended. Future studies should examine change in volume of tobacco sales at liquor stores and tobacco products shops, as well as future comparisons between cities that restrict availability such as those examined in this study, and policies that ban all menthol sales to determine which are more impactful at reducing the health disparities related to menthol tobacco products.

## Conclusion

Advocates successfully countered tobacco industry attempts to misdirect focus from health to profits and charges of criminalization and racism. Passing the policy was just the beginning; it was necessary for advocates to monitor implementation and maintain engagement to address challenges. As efforts by retailers to circumvent the ordinances occurred, the solid base of support built during policy passage helped city council members resist efforts to weaken or overturn ordinances. While each city experienced some implementation challenges, advocates had adequately prepared council members to anticipate challenges, and worked with them to strengthen original ordinances. Their experience may provide useful lessons for advocates and decision-makers in other communities considering policies to reduce availability of menthol tobacco products.

## Abbreviation Used

FDA: Food and Drug Administration

## Notes

^†^ Tobacco in this document refers specifically to the use of manufactured, commercial tobacco products, and not to the sacred, medicinal and traditional use of tobacco by American Indian people and other groups.

^‡^ Tobacco products shop means a retail establishment with an entrance door opening directly to the outside that derives at least 90% of its gross revenue from the sale of tobacco products, loose tobacco, plants, or herbs and cigars, cigarettes, pipes, and other smoking devices for burning tobacco and related smoking accessories and in which the sale of other products is merely incidental. Tobacco products shop does not include a tobacco department or section of any individual business establishment with any type of liquor, food, or restaurant license.
